# SWI/SNF-type complexes–transcription factor interplay: a key regulatory interaction

**DOI:** 10.1186/s11658-025-00704-y

**Published:** 2025-03-10

**Authors:** Anna Maassen, Jaroslaw Steciuk, Magdalena Wilga, Jakub Szurmak, Damian Garbicz, Elzbieta Sarnowska, Tomasz J. Sarnowski

**Affiliations:** 1 Institute of Biochemistry and Biophysics Polish Academy of Sciences, Warsaw, Poland; 2 Maria Sklodowska-Curie National Research Institute of Oncology, Warsaw, Poland; 3 Max Planck Institute for Plant Breeding Research, Cologne, Germany

**Keywords:** SWI/SNF, Chromatin remodeling, Transcription factors, Human, *Arabidopsis*

## Abstract

ATP-dependent switch/sucrose nonfermenting-type chromatin remodeling complexes (SWI/SNF CRCs) are multiprotein machineries altering chromatin structure, thus controlling the accessibility of genomic DNA to various regulatory proteins including transcription factors (TFs). SWI/SNF CRCs are highly evolutionarily conserved among eukaryotes. There are three main subtypes of SWI/SNF CRCs: canonical (cBAF), polybromo (pBAF), and noncanonical (ncBAF) in humans and their functional *Arabidopsis* counterparts SYD-associated SWI/SNF (SAS), MINU-associated SWI/SNF (MAS), and BRAHMA (BRM)-associated SWI/SNF (BAS). Here, we highlight the importance of interplay between SWI/SNF CRCs and TFs in human and *Arabidopsis* and summarize recent advances demonstrating their role in controlling important regulatory processes. We discuss possible mechanisms involved in TFs and SWI/SNF CRCs-dependent transcriptional control of gene expression. We indicate that *Arabidopsis* may serve as a valuable model for the identification of evolutionarily conserved SWI/SNF–TF interactions and postulate that further exploration of the TFs and SWI/SNF CRCs-interplay, especially in the context of the role of particular SWI/SNF CRC subtypes, TF type, as well as cell/tissue and conditions, among others, will help address important questions related to the specificity of SWI/SNF–TF interactions and the sequence of events occurring on their target genes.

**Graphical Abstract:**

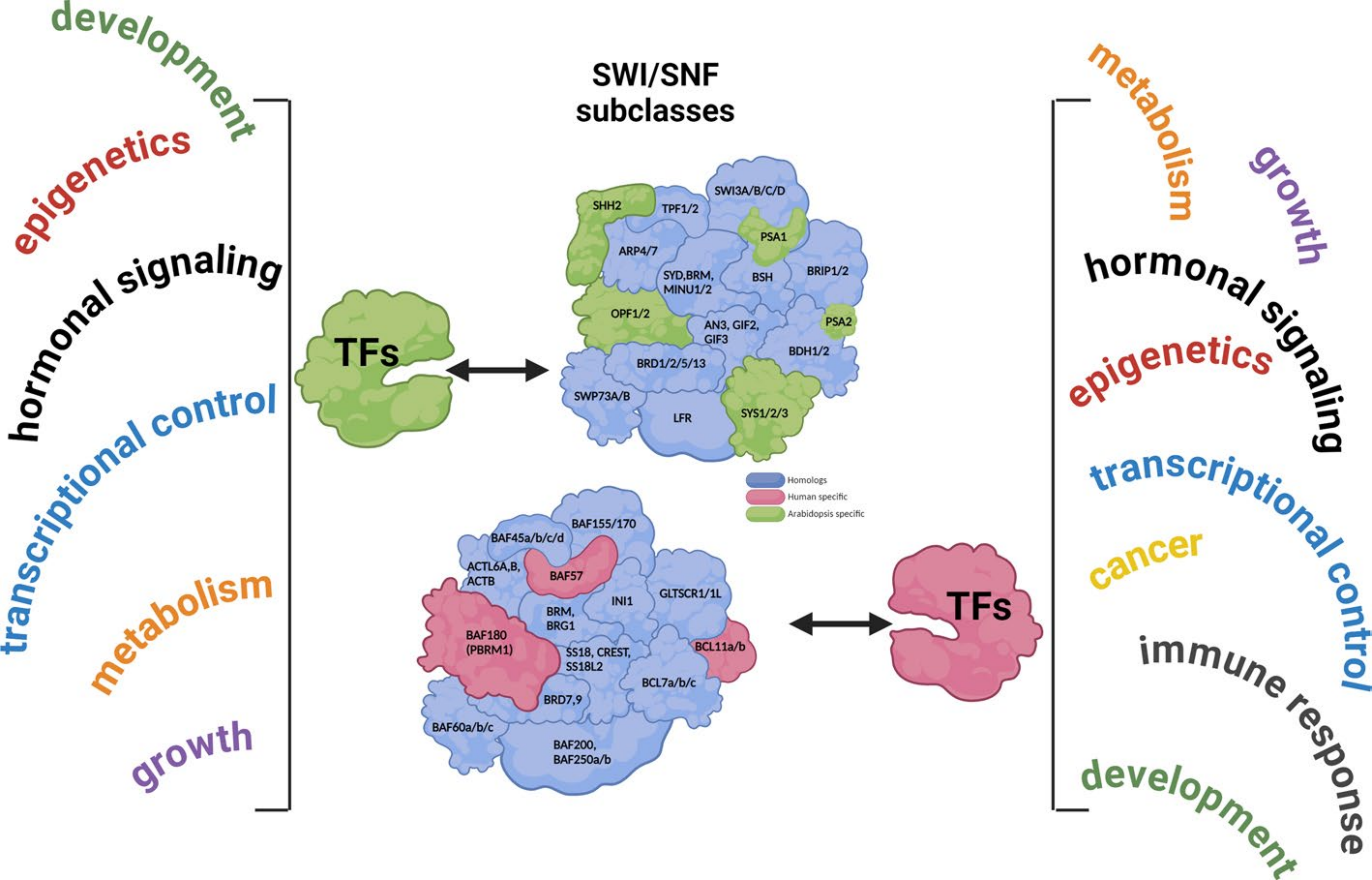

**Supplementary Information:**

The online version contains supplementary material available at 10.1186/s11658-025-00704-y.

## Introduction

The genomes of all eukaryotes encode thousands of genes; however, only a fraction of these are actively transcribed simultaneously. Which genes are active at a particular moment depends largely on factors such as cell type, phase of the cell cycle, and sensed environmental stimuli, among others. Maintenance of proper gene expression patterns is particularly complicated in highly differentiated multicellular organisms such as humans or plants and requires tight and precise controlled cooperation between various molecules. The action of transcription factors (TFs) is one of the most important mechanisms for controlling gene expression. TFs specifically bind to *cis*-acting elements in promoter regions of their target genes and additionally to distal regulatory elements—enhancers. The activity of TFs is often cell-type specific and is additionally influenced by interacting cofactors, binding partners, modifying enzymes, and the local chromatin environment [1]. Their mode of action depends on the molecular context of stimuli, thus affecting the arrangement of the transcriptional complex on a target gene, resulting in activation or repression of gene expression, respectively [2]. The nucleosomal structure of chromatin prevents random binding of circulating TFs. Therefore, specialized mechanisms exist, that dynamically regulate DNA–nucleosome interactions and thus the access to sequences present in DNA. For example, TFs can cooperate with complexes that directly remodel chromatin, using energy derived from ATP hydrolysis, and therefore destabilize interactions between histones and DNA, making DNA accessible for the binding of specific proteins related to transcription and other processes [3].

Here, we summarize current knowledge of TFs interactions with the switch/sucrose nonfermenting (SWI/SNF) subfamily of chromatin remodeling complexes (CRCs) and their implications for higher organisms such as humans and plants. We show that *Arabidopsis thaliana*, a plant, represents a perfect model for studying such human processes as those related to cancer and other diseases. This includes the assessment of TFs and chromatin remodeling regulatory functions given, for example, the existence of viable mutant lines with inactivated subunits of SWI/SNF chromatin remodeling complexes and numerous transcription factors. In addition, it represents relatively easily applicable approaches utilizing simple and cost-effective classical genetics and molecular biology methods as well as reduced restrictions and a lack of ethical issues related to the use of plant models [4–8]. We highlight the existing gaps in the current knowledge and propose new directions for further study to overcome the limitations.

## SWI/SNF–TF interplay and its implication on transcription

**Fig. 1:**
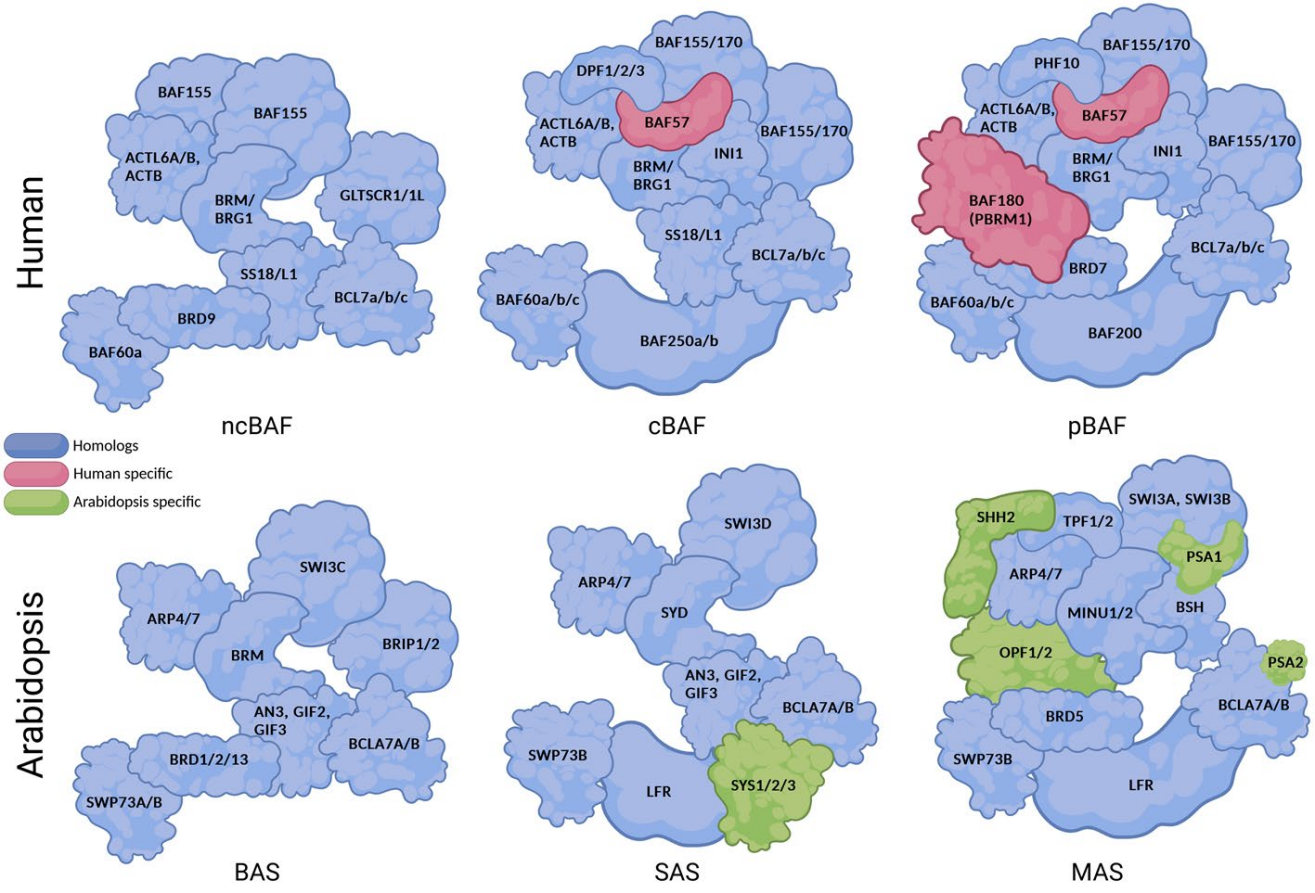
Human and *Arabidopsis* possess three evolutionarily conserved and functionally analogical subtypes of SWI/SNF CRCs

There are four known subfamilies of ATP-dependent chromatin remodeling complexes (CRCs) classified on the basis of the type of their central ATPase subunit: SWI2/SNF2, ISWI, Mi-2 (CHD1), and INO80. The most intensively studied subfamily is switch/sucrose nonfermenting (SWI/SNF), which locally opens chromatin structure by moving or ejecting nucleosomes [9]. The subfamily of SWI/SNF complexes was first discovered in yeast, where two SWI/SNF CRC subtypes exist (ySWI/SNF and yRSC). Subsequent studies proved the subfamily of SWI/SNF CRCs to be highly evolutionarily conserved among eukaryotes, including human and plants—the most complicated organisms in their kingdoms. Three main SWI/SNF subtypes, referred to as canonical (cBAF), polybromo (pBAF) and noncanonical (ncBAF), exist in humans [10, 11], while in *Arabidopsis*, there are the following functional counterparts: SYD-associated SWI/SNF (SAS), MINU-associated SWI/SNF (MAS), and BRAHMA (BRM)-associated SWI/SNF (BAS) (Fig. 1) [12]. SWI/SNF CRC subtypes share common and specific subunits, allowing functional diversification. Given the combinatorial possibilities and subunit exchange, the main classification is not fully exhaustive, as specialized subtypes such as esBAF and nBAF and others may exist in humans and *Arabidopsis*.

Pioneering data [13, 14] serve as a solid foundation for the theory that SWI/SNF complexes facilitate access of TFs to binding sites located within nucleosomes. However, SWI/SNF interactions with DNA and nucleosomes are not sequence specific [15, 16]. This fact, as well as the low abundance of SWI/SNF in the nucleus relative to the number of nucleosomes, suggests that the complex must be targeted to genomic regions where its activity is required [17]. For example, the pBAF complex predominantly controls the chromatin accessibility at promoter regions, while cBAF-type SWI/SNF CRCs control chromatin structure at bivalent promoter regions and distal enhancer regions [18]. By contrast, ncBAF-type SWI/SNF CRCs likely prefer promoter regions enriched in H3K4-trimethylation, topologically associating domain (TAD), and CCCTC-binding factor (CTCF) sites [19].

Of note, the inactivation/impairment of subunits of SWI/SNF CRCs in humans and in *Arabidopsis* has profound effects on transcriptome resulting in both gene expression activation and repression [20]. Although the direct repressive effect of SWI/SNF CRCs on gene expression seemed to be controversial, there is ample solid evidence that rapid inactivation of subunits of SWI/SNF CRCs (e.g., Brahma-related gene 1 (BRG1) ATPase or BAF250a) results in similar numbers of genes activated and repressed [21]; however, the involvement of particular subtypes of SWI/SNF CRCs and the mechanism remain still elusive.

The SWI/SNF CRCs function, among others, relies on TF interactions with these complexes [22]. The interplay between SWI/SNF CRCs and TFs may be based on several different mechanisms; one of them is the interaction of pioneer transcription factors (PTFs) [23]. PTFs are a special class of TFs that are able to access their DNA target sites in closed chromatin, allowing the binding of other TFs. Pioneer TFs have the ability to bind directly to DNA in nucleosomes and are relevant for early regulatory steps in transcription. They can exert positive or negative effects [24] on the transcriptional initiation.

**Fig. 2:**
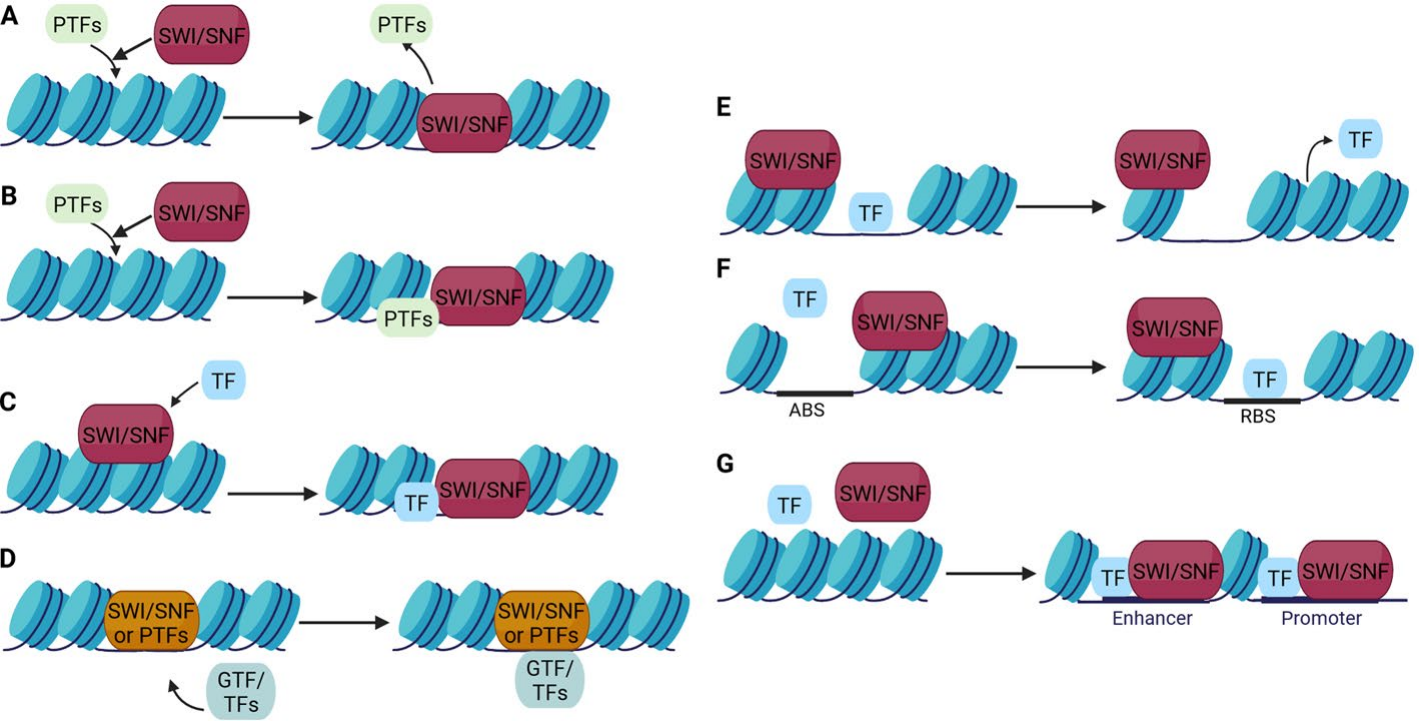
Transcriptional control of gene expression involves interaction between the SWI/SNF CRC and transcription factors. **A** A pioneer transcription factor binds to specific DNA sites and then forms a complex with SWI/SNF to open chromatin. **B** A pioneer transcription factor recruits SWI/SNF to chromatin and increases chromatin accessibility. **C** A TF is recruited by SWI/SNF, which promotes SWI/SNF complex-dependent chromatin modeling. **D** The different modes of action of pioneer TFs, TFs, and general TFs (GTFs) and their interdependence with SWI/SNF chromatin remodeling complexes. **E** Chromatin remodeling by SWI/SNF-induced nucleosome sliding and TF eviction. **F** The SWI/SNF complex activates or silences gene expression by controlling access to binding sites for an activator (ABS) or repressor (RBS) of transcription. **G** The SWI/SNF complex plays an essential role in modulating promotor and enhancer accessibility, required for TF-mediated gene expression activation

PTFs bind to DNA and recruit SWI/SNF CRCs. They can be actively displaced from the chromatin by the SWI/SNF complexes (Fig. 2A) [25]. Alternatively, PTFs, together with SWI/SNF can increase chromatin accessibility (Fig. 2B). The inhibition of the interaction between the classical PTF-GATA3 and SWI/SNF negatively affects the opening of chromatin [25]. SWI/SNF CRCs can actively recruit TFs (Fig. 2C). Additionally, TF activity is modulated by interacting partners—cofactors (coactivators, corepressors) [26]. Cooperative binding of TFs and their coregulators regulates main transcription actions driven by Pol II and a group of general TFs (GTFs) (Fig. 2D) [27]. SWI/SNF-recruited TF may recruit further TFs, histone acetyltransferases, or other chromatin modifiers that promote SWI/SNF-dependent chromatin remodeling at the promoter region [28]. During the remodeling action, the SWI/SNF complex may encounter DNA-binding proteins such as TFs (Fig. 2E) [29]. It has also been shown that SWI/SNF was able to slide a nucleosome past a TF, with concurrent eviction of the TF from the DNA [29]. SWI/SNF CRCs contribute to the dynamic activation or silencing of genes by facilitating or controlling the binding of transcriptional activators or repressors (Fig. 2F) [30]. Different subtypes of SWI/SNF have distinct localization profiles across enhancers, promoters, and gene bodies (Fig. 2G) and their distinctive compositions are thought to provide specificity in interactions with TFs and other chromatin regulators [31].

## SWI/SNF–TF interplay in regulatory processes

Signaling cascades activate a specific set of TFs, generating a particular gene expression profile leading to a final specific cellular response [32].

**Fig. 3:**
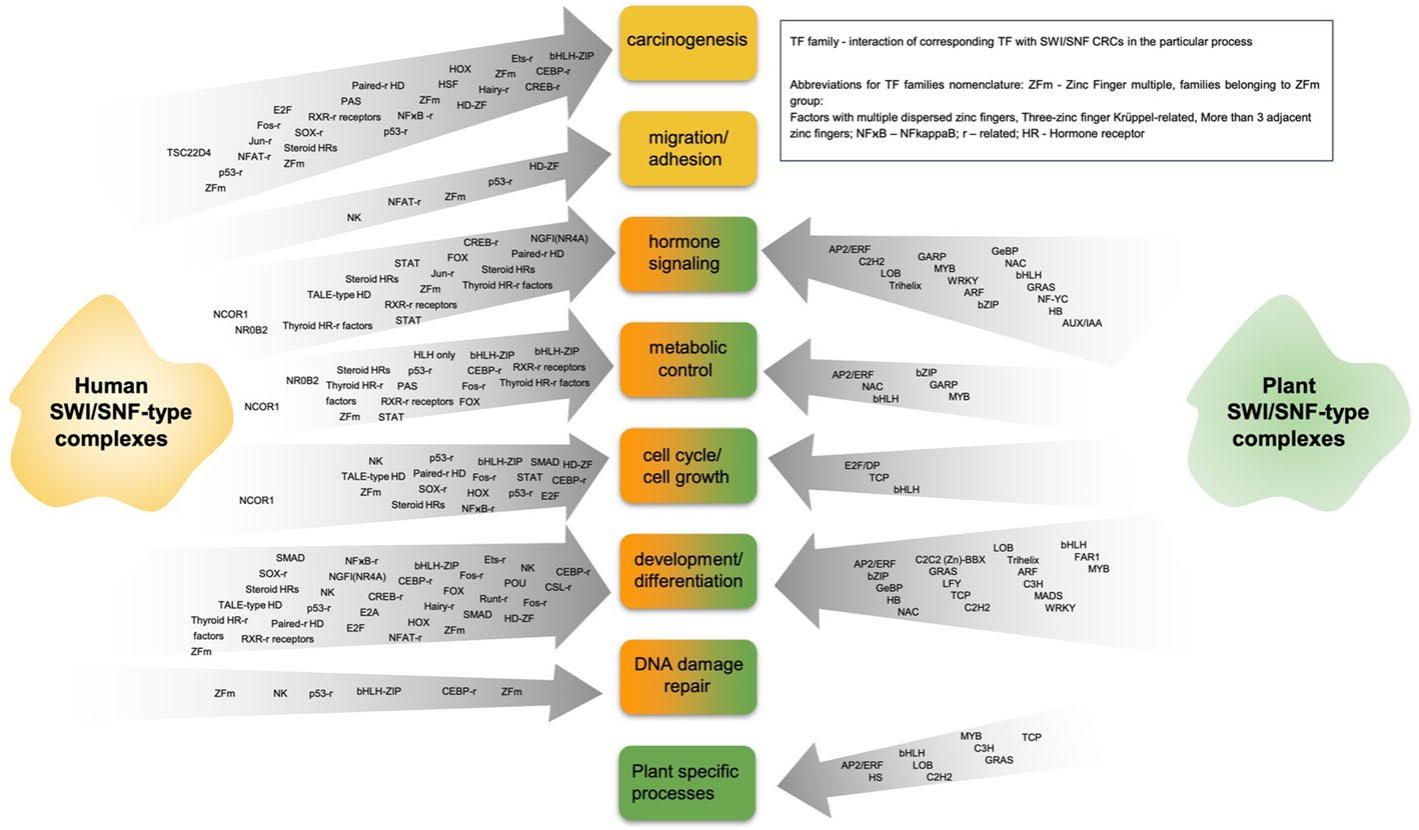
SWI/SNF chromatin remodeling complexes interact with TFs involved in various regulatory processes, and carcinogenesis. Family names for human TFs were found at https://genexplain.com/tfclass/huTF_classification_Genera.html or http://tfclass.bioinf.med.uni-goettingen.de/ and for plant TFs and cofactors at http://itak.feilab.net/cgi-bin/itak/db_browse.cgi

A detailed search of interactors of subunits of human SWI/SNF CRCs using the BIOGRID database resulted in the identification of a group of TFs and their cofactors and processes for which SWI/SNF–TF interactions are required. These processes may be grouped into general classes such as carcinogenesis, migration and adhesion, hormone signaling, metabolic control, cell cycle, cell growth, development and differentiation, and DNA damage repair, among others. (Fig. 3; Supplementary Table 1, Subtable 1). A search of the ENCODE database [33] of Chromatin immunoprecipitation next-generation sequencing (ChIP-seq) experiments revealed the presence of SWI/SNF CRC subunits on most promoters of genes encoding partner TFs for SWI/SNF, suggesting the existence of an additional controlling feedback loop between TFs and SWI/SNF (Supplementary Table 1, Subtable 2).

After the release of the entire *A. thaliana* genome sequence, it was determined that around 1500 of the 25,498 genes were coding for transcription factors [34]. About half of all *Arabidopsis* TFs are determined to be plant-specific, while other transcription factors are evolutionarily conserved between kingdoms [35]. Most members of plant-specific TF families characterized so far are involved in the regulation of genes related to the development of organs specific to plants, and the response system for adaptation to terrestrial environments [36].

As in humans, the transcription process in plants involves chromatin remodeling, conducted particularly by SWI/SNF CRCs. Interactions between SWI/SNF CRCs and TFs also occur in plants. A yeast two-hybrid interactome study revealed numerous TFs as SWI/SNF partners [37–39]. All transcription factors known to interact with SWI/SNF subunits so far in *Arabidopsis* can be grouped into 34 different families. Among them, basic helix–loop–helix (bHLH), myeloblastosis (MYB), APETALA2/ethylene-response factor (AP2/ERF)-ERF, basic leucine zipper (bZIP), teosinte branched 1, cycloidea, and proliferating cell factor (TCP) are the most strongly overrepresented, according to classification by the iTAK database (Fig. 3; Supplementary Table 1, Subtable 3) [40].

In addition to physical interactions, as in humans, plant SWI/SNF CRCs also seem to have a significant role in the regulation of the expression of TF encoding genes. Two independent studies have shown that BRM ATPase is located on a large number of promoters of all SWI/SNF-interacting TF genes thus far identified in *Arabidopsis* (Supplementary Table 1, Subtable 4) [41].

On the basis of the available data, it can be concluded that both human and plant SWI/SNF CRCs make up a complex network of interdependence with TFs. This network regulates a wide range of general and some plant-specific processes (*Arabidopsis*) as well as carcinogenesis in human (Fig. 3).

### Development and differentiation

There are no data on the effect of full inactivation of SWI/SNF CRC subunits on human development. It may be likely due to the lethality at the early developmental stages, as is observed in the case of mice. Even the point mutations in genes encoding certain subunits, e.g., ATPase or BAF250 in humans, lead to the development of various syndromes such as Coffin–Siris syndrome, Nicolaides–Baraitser syndrome, or nonsyndromic intellectual disability [42], while the loss of heterozygosity of the *SMARCB1* gene encoding the INI1 subunit leads to the development of malignant rhabdoid tumors in children [43].

The study on *Arabidopsis* indicated that the inactivation of SWI/SNF CRC subunits leads to lethality at the early stage of development; however, in some cases, the mutant lines were viable but presented numerous developmental alterations [20, 44]. The existence of viable mutants provided a unique possibility for the investigation of the effects of SWI/SNF CRC subunits inactivation at the level of the whole organism. Moreover, the use of plant lines is subject to fewer restrictions than the use of animals or human-derived tissues and generates no ethical issues, thus representing a unique opportunity for studying evolutionarily conserved functions of SWI/SNF CRCs between plant and animal kingdoms.

Interactions of human SWI/SNF complexes and RUNX family transcription factor 1 (RUNX1) were shown to be relevant during myeloid differentiation through transcriptional regulation [45]. The BRG1 and INI1 subunits of SWI/SNF CRCs associate with RUNX1 and are recruited to promoters of RUNX1 hematopoietic target genes encoding granulocyte–macrophage colony-stimulating factor (GM-CSF) and interleukin 3 (IL-3) cytokines, MCSF-R cytokine receptor, or p21 cyclin-dependent kinase inhibitor. These interactions with cytokine promoters correlate with histone modifications characteristic of active chromatin. Downregulation of RUNX1 reduces the binding of BRG1 and INI1 to these promoters. Decreased association of RUNX1 and SWI/SNF CRC subunits to the *GM-CSF* and *IL3* promoters correlates with reduced expression of these genes, indicating that RUNX1 supports recruitment of BRG1 and INI1 to target gene promoters to regulate their expression [45].

Similarly, BRG1-recruitment for transcriptional activation of the *SLC11A1* gene (also known as natural-resistance-associated macrophage protein 1) was shown during the differentiation of HL-60 promyeloblast cells toward macrophages [46]. Studies of the mechanism of transcriptional activation of the *SLC11A1* promoter have shown its dependency on the cooperation of ATF-3 TF with BRG1 and β-actin.

Analysis of the polycomb group ring finger 1 (PCGF1) protein interacting partners in the embryonal carcinoma cell line NT2—a model for neuronal differentiation [47]—revealed the interaction of two subunits, BAF170 and BAF250b, with components of a pluripotency protein subnetwork. Additionally, BAF170 has been shown to interact with the POU class 5 homeobox 1 (POU5F) TF and with the developmental pluripotency associated 4 (DPPA4) cofactor, whereas BAF250b, a characteristic subunit of cBAF, has been shown to interact with two TFs—nanog homeobox (NANOG) and POU5F. However, the consequences of their interaction with cBAF and potentially the pBAF subtype of SWI/SNF CRCs for gene expression regulation were not explored [47].

Another differentiation process in which interactions of SWI/SNF CRC subunits and TFs were identified is the development of the human heart. Interaction during cardiogenesis between GATA4 and BAF155 was reported. This interaction is supposed to play a role in human congenital heart malformations [48]. The BAF250a subunit of cBAF was found to be capable of coordinating cardiogenesis [49]. In a study using cultured human embryonic stem cells, the interaction with proteins TBXT (which regulates mesoderm formation) and MEF2C (a key cardiac cardiomyogenic TF) was found. Interestingly, this study noted opposite roles of BAF250a in governing cardiogenesis and neurogenesis—it promoted cardiogenesis while inhibiting neurogenesis by interacting with REST TF, suggesting a dual role of the cBAF complex in these processes. The involvement of genes responsible for neurogenesis in transcriptional repression, through interaction with REST, has also been observed for BRG1 and BAF170 subunits of SWI/SNF CRCs [50]. Furthermore, the PTFASCL1 interacts with BAF155 and BAF250a subunits to control chromatin accessibility at neurogenic loci to coordinate neurodifferentiation indicating a possible role of cBAF subtype in this process [51].

**Fig. 4:**
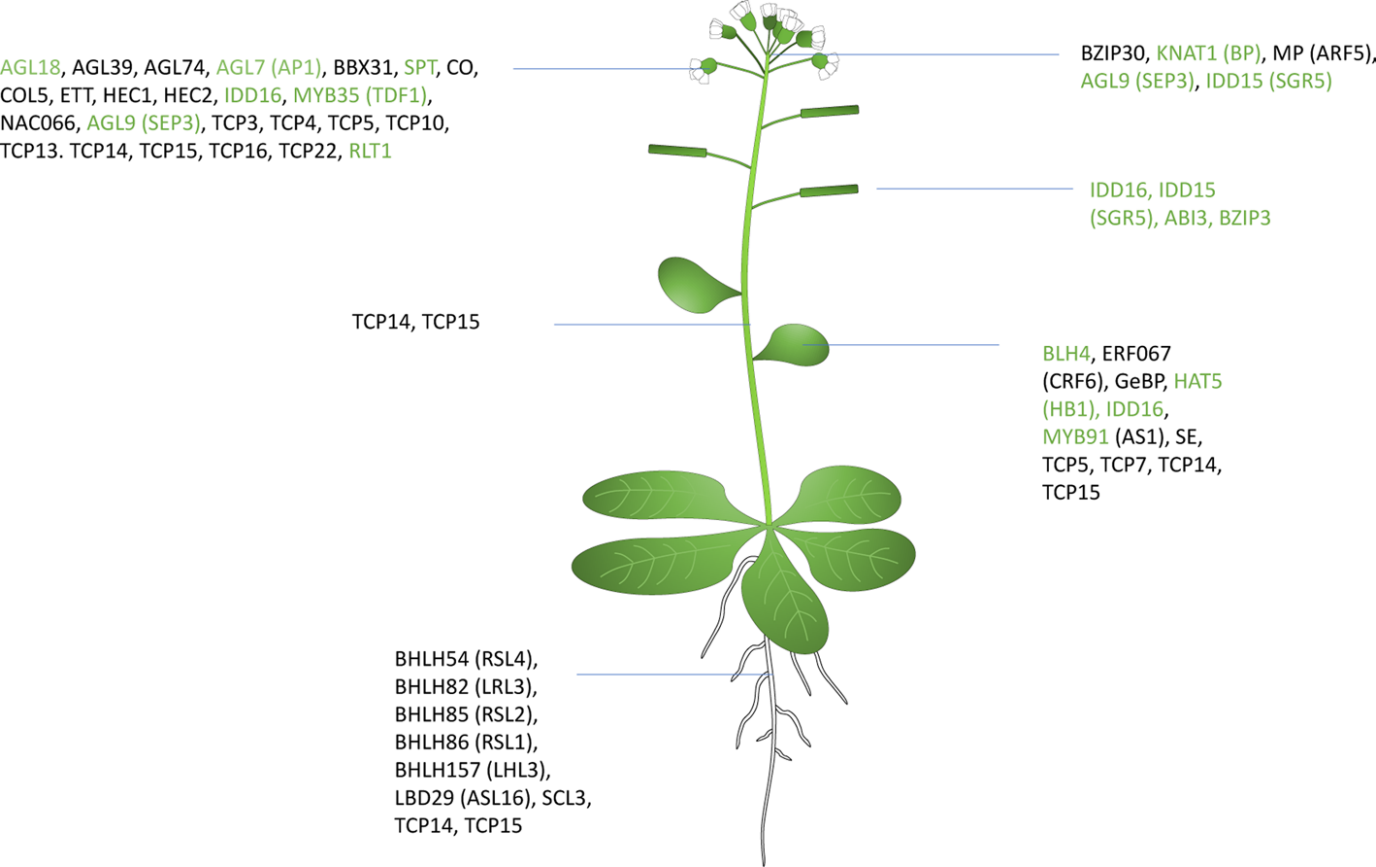
Transcription factors interacting with the plant SWI/SNF CRCs have an important role in development. Green-colored TFs are homologous to human TFs

The study on *Arabidopsis* indicated that SWI/SNF CRCs play an important role in development and differentiation, and more than 100 TFs have been suggested to interact with SWI/SNF CRC subunits [37, 38]; among these, numerous TFs share high homology with human TFs (Fig. 4). It has been shown that BRM and SYD ATPase subunits of *Arabidopsis* SAS and BAS subtypes of SWI/SNF CRCs interact with the MONOPTEROS (MP) transcription factor involved in inflorescence development and bind to its critical targets [52]. Upon auxin sensing, SWI/SNF ATPases are recruited by MP, increasing DNA accessibility for induction of key regulators of flower primordium initiation. When the hormonal signal is missing, auxin-sensitive Aux/IAA proteins remain bound to MP, blocking recruitment of SWI/SNF ATPases and recruiting a corepressor TOPLESS (TPL), which interacts with histone deacetylase HDA19, generating a barrier to a transcriptionally active chromatin state.

The SWI/SNF complexes also play a role in subsequent stages of inflorescence development, contributing to shaping its proper architecture through cooperation with the BREVIPEDICELLUS (BP/KNAT1) protein [53] belonging to the class I KNOTTED-1 homeobox (KNOX) transcription factor family. BP physically interacts with BRM ATPase and targets both *KNAT2* and *KNAT6* (class I *KNOX* genes) in a BP-dependent manner. Moreover, yeast two-hybrid data imply the interaction of KNAT1 with SWI3B [37], collectively indicating involvement of both *Arabidopsis* BAS and MAS subtypes of SWI/SNF CRCs in these processes.

BRM and SYD, as well as SWP73B, co-purify in complexes with floral identity-controlling homeotic proteins AGAMOUS (AG), SEPALLATA3 (SEP3), APETALA1 (AP1), and APETALA3 (AP3) [54]. BRM, SYD, and SWP73B were especially highly enriched in the AP1 immunoprecipitated fraction [20, 54], indicating a role of BAS and SAS subtypes of SWI/SNF CRCs in the floral identity control [55].

SWI/SNF complexes have also been shown to be able to overcome polycomb repression of AG and AP3 [56]. BRM and SYD ATPases physically interact in plant cells, with SEP3 and LEAFY (LFY) considered as plant PTFs [57]. BRM and SYD are recruited to AP3 and AG regulatory regions during flower development. At least in the case of SYD, this recruitment has been shown to be LFY and SEP3-dependent. BRM and SYD are redundantly required for AP3 and AG expression activation and patterning of flower three central whorls, and this requirement can be overcome by the reduction of polycomb repression, indicating a role of both BAS and SAS SWI/SNF CRCs and polycomb in this process [56].

The BAS subtype of SWI/SNF CRCs has a role in fine-tuning flowering time. BRM was found to physically interact with the GNC TF and bind to the locus coding for SOC1 protein [58]. The significance of this observation is reflected in the fact that both *brm* and *gnc* mutants show decreased levels of SOC1 transcript, which is associated with the increase of histone H3 lysine 4 trimethylation level and with decreased DNA methylation levels. This indicates that together with GNC, BRM acts as a repressor of SOC1. Moreover, BRM, together with REF6, appears to form a complex with SOC1 itself that relaxes and opens chromatin at the gene encoding TFS1, another TF contributing to the control of proper flowering time. This facilitates the binding of SPL9 protein and activates poised RNAPII, which results in a reduction of the H3K27me3 level across the whole *TFS1* locus [59].

*Arabidopsis* SWI/SNF CRC-TFs interactome data provide a foundation for studying these interactions in human cells for evolutionarily conserved TFs (Fig. 4).

### Control of the cell cycle

SWI/SNF CRCs have been shown to be involved in cell cycle control in both humans and plants, owing to their interaction with cell division-associated TFs and corepressors. In humans, BRG1 ATPase interacts with E2F6, a member of the E2F transcription factor family [60] implicated in activities linked to G1/S and G2/M cell cycle phase transition [61]. It has been found that BRG1 containing SWI/SNF CRCs, in concert with E2F6, may have a function in the regulation of the G1/S-related gene expression in the cell cycle [60]. Additionally, the presence of the transcriptional repressor HIC1, which interacts with BAF250a on the *E2F1* promoter, further supports the importance of SWI/SNF–TF interactions in the control of cell cycle-associated gene expression [62].

Mechanisms directly regulating E2F1 activity are associated with the retinoblastoma protein (RB1) cofactor, which is critical for cell cycle control. BRM was found to cooperate with RB1 to repress E2F1 activity and thus induce G1 arrest [63]. RB1 was shown to form a repressor complex with histone deacetylase (HDAC) and the SWI/SNF CRCs, thereby regulating transcription of genes encoding cyclins A and E during the cell cycle, and in turn, controlling the exit from G1 and S phases [64].

Interactions of the SWI/SNF CRCs and SMAD TFs were also observed in transforming growth factor beta (TGFβ) signaling [65, 66]. TGFβ regulates cell proliferation, differentiation, and growth. TGFβ modulates the expression and activation of other growth factors [67] and induces epithelial-to-mesenchymal transition (EMT) and cell migration [68], although there are no data on which SWI/SNF CRC subtype is involved in this regulatory process.

In plants, SWI/SNF CRC subunits were found to physically interact with E2FA and E2FD, as well as with E2F dimerization partner DPa [38], suggesting that this complex may be involved in the RBR/E2F pathway, which regulates G1/S transition. Furthermore, *Arabidopsis* SWI/SNF subunits bind TCP14, TCP15, and TCP20 transcription factors [37, 38]. TCP14 and TCP15 were shown to regulate the balance between cell endoreduplication and mitosis by affecting the expression of key cell cycle regulators RBR and CYCA2-3 [69]. TCP20, however, binds the promoter of cyclin CYCB1-1 [70]. Moreover, together with transcription factors, SWI/SNF CRCs may take part in the regulation of meiosis by interacting with FST [37], a TF required for the initialization of meiotic synchrony and normal meiotic entry [71].

These findings highlight strong evolutionary conservation of interaction between TFs and SWI/SNF CRCs human and *Arabidopsis* in the maintenance of proper cell cycle.

### Hormonal signaling

Hormone-dependent interaction between the estrogen receptor (ER) and ATPase (BRM and BRG1) subunits of SWI/SNF CRCs has been demonstrated in human cells. BRG1-mediated coactivation of ER signaling in the cell line SW13 was regulated by the state of histone acetylation—inhibition of HDAC activity by trichostatin A. This resulted in a great increase in BRG1-mediated coactivation, while overexpression of HDAC-1 significantly reduced it, thereby providing an additional level of regulation [72].

BRG1 has also been shown to play a role in glucocorticoid negative feedback regulation of the proopiomelanocortin (*POMC*) gene, which plays a critical role in the hypothalamic–pituitary–adrenal axis [73]. BRG1 participates in the molecular mechanism of *trans*-repression between the NR3C1 glucocorticoid receptor (GR) and the NR4A2 steroid-thyroid hormone-retinoid receptor (NGFI-B), involved in *POMC* expression control. BRG1 has been shown to be critical in the formation of stable in vivo complexes between GR and NGFI-B and between GR and HDAC2. Promoter recruitment for both GR and HDAC2 is glucocorticoid-dependent and is associated with the reduction of acetylated histone H4 resulting in inhibition of transcription initiation. The high frequency of misexpression of BRG1 or HDAC2 in human glucocorticoid-resistant corticotroph adenomas, characteristic of Cushing’s disease, indicates the relevance of these proteins in glucocorticoid-dependent negative feedback regulation and in mechanisms of resistance to this hormone.

BRM interactions with hormonal signaling pathways have mostly been observed with steroid hormones, with responses both in hormone synthesis and at the receptor—it has been shown that alterations in BRM may affect steroid hormone synthesis in prostate cancer [74]. Moreover presence of both BRM and BRG1, as prohibitive corepressors, is required for growth-suppressing signaling by estrogen antagonist [75].

Another subunit of SWI/SNF CRCs, BAF60a, is required for glucocorticoid receptor-dependent chromatin remodeling during transcriptional activation [76]. BAF60a has been shown to mediate the interaction of the NR3C1 glucocorticoid receptor (GR) with BRG1-containing SWI/SNF complexes. Stable expression of a truncated BAF60a mutant protein in UL3 human osteosarcoma cell lines led to disruption of the interaction between GR and the BRG1-containing SWI/SNF CRC. This led to disruption of GR-dependent chromatin remodeling, and subsequently, disruption of transcription. BAF60a also interacts with other hormone receptors such as progesterone receptors (PGR) and estrogen receptors (ER).

Additionally, BAF60a partakes in hormone-dependent interactions with androgen receptors (AR). In the prostate carcinoma LNCaP cell line, BAF60a has been shown to interact directly with the coactivator groove in the AR ligand-binding domain [77]. This interaction with AR influences the expression of androgen-responsive genes. BAF60a appeared to be essential for high AR-dependent *TMPRSS2* expression, as it was almost completely blocked when BAF60a was depleted.

SWI/SNF CRCs are also involved in the progesterone signaling [78]. Presence of SWI/SNF complex is necessary for the induction of progesterone target genes such as *FOS* and *MYC*. Moreover, BAF250a and BAF57 subunits exhibit a hormone-dependent interaction with PGR. After hormone treatment, BRG1, BRM, BAF250a, and BAF57 subunits were shown to be recruited to the mouse mammary tumor virus (MMTV) promoter, which, together with PR, suggests a role of SWI/SNF CRCs in this process.

The *Arabidopsis* SWI/SNF CRCs have been shown to play a role in cytokinin-dependent regulation of leaf maturation. BRM and SWI3C subunits of the BAS subtype of SWI/SNF CRCs physically interact with the TCP4 protein, which belongs to a family of noncanonical bHLH TFs [38]. Such TFs reduce the cytokinin sensitivity of leaves. TCP4 and BRM together bind regulatory regions in *Arabidopsis* leaf cells, including the promoter of the *ARR16* gene, coding for an inhibitor of cytokinin response.

Another function of SWI/SNF CRCs resulting from interaction with TFs is the regulation of response to abscisic acid (ABA) and salinity. BRM and other subunits of SWI/SNF CRCs were shown to interact with ERFVII transcription factors [38, 79]. ERFVIIs positively regulate ABA sensitivity and seed dormancy by binding to the ABSCISIC ACID INSENSITIVE 5 (ABI5) TF promoter in a double GCC element also recognized by BRM [80]. The seedlings of *brm erfVII* sextuple mutants show decreased survival in response to high salinity and increased ABA tolerance by seedling roots, providing evidence for the biological importance of this interaction [79].

Numerous other transcription factors and coactivators regarded as hormone signaling regulators have been identified as probable SWI/SNF CRCs interactors in screen studies [37, 38]. Those include TFs mediating GA, ABA, auxin, jasmonate, ethylene, cytokinin, and brassinosteroids signaling, suggesting an important role in their interaction with SWI/SNF in hormone-mediated signal transduction.

In another study, it has been shown that the *Arabidopsis* functional ERECTA and its human functional counterpart HER2 (epidermal growth factor receptor family member) play a noncanonical role in the transcriptional control of some genes. The SWI3B subunit of the *Arabidopsis* MAS complex has been shown to bind the loci of *GID1* gibberellin hormone receptor encoding genes together with the ERECTA epidermal patterning factor receptor. A similar interaction has been shown for human HER2 and BAF155 [81].

### Metabolic processes

In conjunction with TFs and corepressors, SWI/SNF CRCs have been shown to have a regulatory impact on the control of metabolic processes. BRG1 forms a transcriptional complex with TF nuclear receptor heterodimer (consisting of nuclear receptor subfamily 1 group H member 2 (NR1H2) and retinoid X receptor alpha (RXRA)). This complex mediates the control of high-density lipoprotein (HDL) metabolism, by influencing the expression of its key regulator—ATP binding cassette subfamily A member 1 gene (*ABCA1*) [82]. ABCA1 is a membrane transporter that participates in phospholipid transfer to apolipoproteins initiating the formation of HDLs [83]. The recruitment of BRG1 to the DR-4 element of the ABCA1 promoter, and the physical interaction of LXR/RXR and BRG1, has been shown to be essential for activation of the ABCA1 promoter.

SWI/SNF CRCs cooperate with the SIN3A/HDAC2 corepressor complex and PRMT5 (type II arginine-specific methyltransferase)in transcriptional repression of the MYC target gene carbamoyl-phosphate synthetase 2, aspartate transcarbamylase, and dihydroorotase (*CAD*), which encodes an enzyme catalyzing biosynthesis of pyrimidine nucleotides [84]. BRG1, SIN3A/HDAC2, PRMT5, and MYC are all directly recruited to the *CAD* promoter. Interactions of MYC, SIN3A/HDAC2-PRMT5, and SWI/SNF CRCs with the promoter are mutually exclusive and cell cycle-dependent. Direct interactions of cofactor SIN3A and MYC with BRG1, and their presence on the *CAD* promoter, suggest a contribution of these interactions to the regulation of *CAD* gene expression by SWI/SNF-dependent chromatin remodeling, in combination with histone modifications.

HDAC1 and SIN3A together form a corepressor complex that also cooperates with SWI/SNF CRCs and is responsible for the regulation of expression of the cholesterol 7 alpha-hydroxylase gene (*CYP7A1*) by bile acids in HepG2 hepatoma cells, a critical step in the maintenance of cholesterol homeostasis [85].

The BAF155 subunit of SWI/SNF CRCs was identified as a regulator of skeletal muscle metabolism in a mouse model [86]. Muscle-specific ablation of BAF155 results in the increase of oxidative metabolism (decreased lactate production and increased intramuscular ATP production) in hypoxia conditions during endurance exercise. Direct interaction of BAF155 with STAT3 was reported, which forms a coactivator complex with HIF-1α to fully activate HIF-1α signaling and induce expression of its target genes regulating glycolytic metabolism.

Several more TFs with roles related to metabolic control have been identified as physical interactors of *Arabidopsis* SWI/SNF CRC subunits [37, 38]. For example, MYB122 is a TF that acts in the biosynthesis of indolic glucosinolates and camalexin [87], NAC032 represses anthocyanin biosynthesis during stress response [88] and increases sugar and amino acid catabolism during carbon starvation [89], and finally, RAP2.2 takes part in the control of carotenoid biosynthesis [90].

### DNA damage repair

Among others, some SWI/SNF CRC subunits were shown to be relevant in response to DNA double-strand breaks (DSBs) as homologous recombination (HR)-promoting proteins [91]. Interactions of the bromodomain-containing BRM ATPase with TFs LBX1, VAX2, ZIC2, ZKSCAN2, and ZNF212 were recognized. The precise processes were not studied in detail, but the interaction of SWI/SNF CRC subunits with protein complexes directly participating in DNA repair was detected [92]. BRG1 was also recognized as an important factor in DBS repair [93]. In the human osteosarcoma cell line U2OS, it has been shown that E4F1 TF binds to BRG1 and is recruited to DNA lesions in a PARP-dependent manner, in support of DNA repair.

Although SWI/SNF complexes were shown to play a role in DNA damage repair in *Arabidopsis* [94], the evidence that their contribution to this process can result from interactions with transcription factors is currently lacking.

### Plant-specific response processes

As sessile organisms, plants face myriad environmental stresses that can significantly impact their growth, development, and survival. In response to these challenges, plants have evolved a diverse range of adaptive mechanisms to ensure their continued existence. Among these mechanisms, chromatin remodeling complexes and transcription factors play pivotal roles in orchestrating rapid and precise gene expression changes.

The role of the interaction between these components has been demonstrated in response to cold tolerance. LFR, a subunit of SAS and MAS SWI/SNF complexes in *Arabidopsis*, has been shown to directly interact with ICE1 [95], a bHLH transcriptional activator of *CBF* genes acting in anti-freezing response. Mutation of LFR resulted in hypersensitivity to freezing stress. Similar observations were made for SWI3C, which also localizes at the promoter of several low-temperature signaling genes, including *ICE1* [96] indicating the role of the BAS subtype of SWI/SNF CRCs in this process. Furthermore, SWI3C interacts with other TFs involved in cold stress response, such as MYB15, MYB96, or CBF4, further supporting the role of BAS subtype [38].

*Arabidopsis* BRM is involved in the control of chlorophyll biosynthesis [97]. Yeast two-hybrid data suggest that BRM and SWI3D also interact with GLK1 [37, 38], another transcription factor involved in chlorophyll biosynthesis [98], indicating the role of BAS and SAS subtypes of SWI/SNF CRCs and TF interactions in this process.

Many other TFs regulating the response to various biotic and abiotic stresses have been shown to interact with SWI/SNF CRC components [38]. However, the significance of these interactions requires further examination.

### Control of cell migration/adhesion

The interaction between BRG1 and the TF specificity protein 1 (SP1) has been shown to be implicated in the expression of the matrix metallopeptidase 2 (*MMP2*) gene, which is involved in cell migration and tumor invasiveness [99]. BRG1 has been shown to regulate *MMP2* expression in SW13 and SK-MEL5 cells by transcriptional control involving SP1 [99]. The modulation of expression of this extracellular matrix remodeler by BRG1 has been shown to be associated with increased invasive ability of melanoma in vitro [99]. Other BRG1 interactors with migration-associated activity are the transcription factor NFATC2 and corepressors NOTCH1 and YAP1.

In cooperation with TFs, the SWI/SNF CRCs are also involved in the transcriptional regulation of proteins associated with cell adhesion. BRG1 interaction with SMAD2/3 TFs has been observed in HaCaT keratinocyte cells, on the promoter of the *CTGF* gene (connective tissue growth factor) responsible for cell adhesion. Recruitment of BRG1 to the *CTGF* promoter was SMAD dependent, and additionally, knockdown of BRG1 substantially decreased recruitment of RNA polymerase II to the CTGF promoter [65].

### Carcinogenesis

Numerous interactome studies in cancer cell lines report the interaction of SWI/SNF CRC subunits with TFs such as MYC [58, 100], SOX2 [101], SOX4 [100], TP53 [102], HOMEZ, HSFY1, IKZF3 [103], JUN [104], EPAS1 [105], ESR1 [106], ESR2 [107], RELB [108], and RXRA [103], although the data highlighting how this interaction may be involved in cancer development is missing.

One of the elucidated mechanisms of the involvement of SWI/SNF CRCs in carcinogenesis is their interaction with aberrant TFs—oncoproteins fused with two members of the FET family of RNA-binding proteins (FUS/EWS/TAF15) [109]. These oncoproteins are known to participate in the regulation of transcription, RNA processing, and RNA transport [110, 111].

The interaction of two chimeric TFs—DDIT3 or FLI1, fused to FUS or EWSR1 FET proteins, with BRG1 containing SWI/SNF complexes—has been shown [109]. Both fusion proteins FUS-DDIT3 and EWSR1-FLI1 were shown to interact with the SWI/SNF CRCs in MLS 402-91 (myxoid liposarcoma) or EWS TC‐71 (Ewing sarcoma) cell lines, with each line carrying different FET oncogenes with simultaneous lack of expression of normal DDIT3 or FLI1. The interactions of DDIT3 with the SWI/SNF CRC subunits BAF155 and INI1 in the myxoid liposarcoma cell line were confirmed [112]. It was also revealed that FET oncoproteins interact with the transcriptional coactivator BRD4 via the SWI/SNF complex, co‐localizing on chromatin, potentially together with mediator and RNA polymerase II, which may be considered as a potential molecular mechanism for the FET‐fusion‐induced oncogenic transcriptional profiles. Since forced expression of FET oncogenes caused an increase of global H3K27 trimethylation levels and an alteration of gene expression patterns in human HT1080 sarcoma cells, a shift in the antagonistic balance between SWI/SNF and PRC2, known to influence H3K27 trimethylation [113], was also proposed as a carcinogenic mechanism. In contrast, the cooperation of SWI/SNF CRC classes containing BRG1 and BRM with the main PRC2 subunit EZH2 was also shown in the control of *PD-L1* gene expression during cancer-induced CD4 + T cell exhaustion [114].

A study revealed that the shortening of the AR (N-terminal poly(Q) tract) increases prostate cancer risk by enhancing androgen-dependent transcriptional activity. This alteration changes AR’s ligand-induced conformation, making it responsive to lower androgen levels [115]. The shortened poly(Q) AR associates with higher levels of p160 coactivators GRIP1, RAC3, and BRG1 and BAF155 SWI/SNF subunits, suggesting enhanced recruitment of coactivators and chromatin remodeling complexes [116–118]. Another study highlighted CEBPA’s role in liver tumor proliferation via posttranslational modification affecting SWI/SNF interactions. CEBPA inhibits CDKs and represses E2F to control hepatocyte proliferation [117]. Phosphorylation at Ser193 is crucial for its growth-inhibitory function. PI3K/Akt activation increases PP2A activity, dephosphorylating CEBPA, disrupting its interaction with cdk2, cdk7, E2F4, and BRM, leading to uncontrolled proliferation. Phosphorylated CEBPA binds BRM and cdk2 to inhibit proliferation, while dephosphorylation sequesters RB1, accelerating growth [118].

Cancer treatment studies highlight the role of TF and cofactor interactions with SWI/SNF CRCs in drug resistance and sensitivity. BRG1 and prohibitin (PHB) are essential for androgen antagonist-mediated repression in prostate cancer [119], while PHB-SWI/SNF interactions aid estrogen antagonist-induced growth suppression in breast cancer [75]. IKZF3-SWI/SNF interaction mediates lenalidomide resistance in myeloma [120]. Sanchez-Tillo and colleagues reported the involvement of BRG1 and ZEB1 interactions in epithelial-to-mesenchymal transition (EMT), promoting tumor invasiveness [121]. ZEB1 plays a role in the control of the expression of key regulatory genes in embryonic development and cell differentiation processes [122]. Expression of ZEB1 leads to EMT and promotes metastasis by repressing calcium-dependent E-cadherin via binding to its promoter region [123, 124]. The loss of E-cadherin is a key initial step in the EMT process. ZEB1 was shown to interact with BRG1 to repress the E-cadherin expression [121]. Blocking the ZEB1–BRG1 interaction induces expression of E-cadherin and downregulation of vimentin—an EMT marker.

SWI/SNF CRCs suppress tumors by interacting with TP53, regulating its target gene expression [125–127] across multiple cancer cell lines. Studies on INI1 and BRG1 show that SWI/SNF CRCs are essential for TP53-mediated apoptosis and growth suppression in sarcoma cell lines. BAF60a acts as a bridge between TP53 and SWI/SNF CRCs [126], with its N-terminal region interacting with TP53’s tetramerization domain. Disrupting this interaction via a dominant-negative BAF60a or siRNA silencing impairs TP53’s antitumor functions, including apoptosis and cell cycle arrest. In gynecological cancers, BAF250a acts as a negative cell cycle regulator and a tumor suppressor gene cooperating with TP53 [127]. Restoring wild-type BAF250a in ovarian cancer cells suppresses proliferation and tumor growth, while its silencing enhances tumorigenicity. Gene expression analysis identified CDKN1A (p21) and SMAD3 as key BAF250a downstream targets [128]. TP53 interacts with BAF250a and BRG1, forming a complex that binds CDKN1A and SMAD3 promoters. Mutations in BAF250a or TP53 are mutually exclusive in tumors and could result in the loss of transcriptional regulation of CDKN1A and SMAD3 [127].

**Fig. 5:**
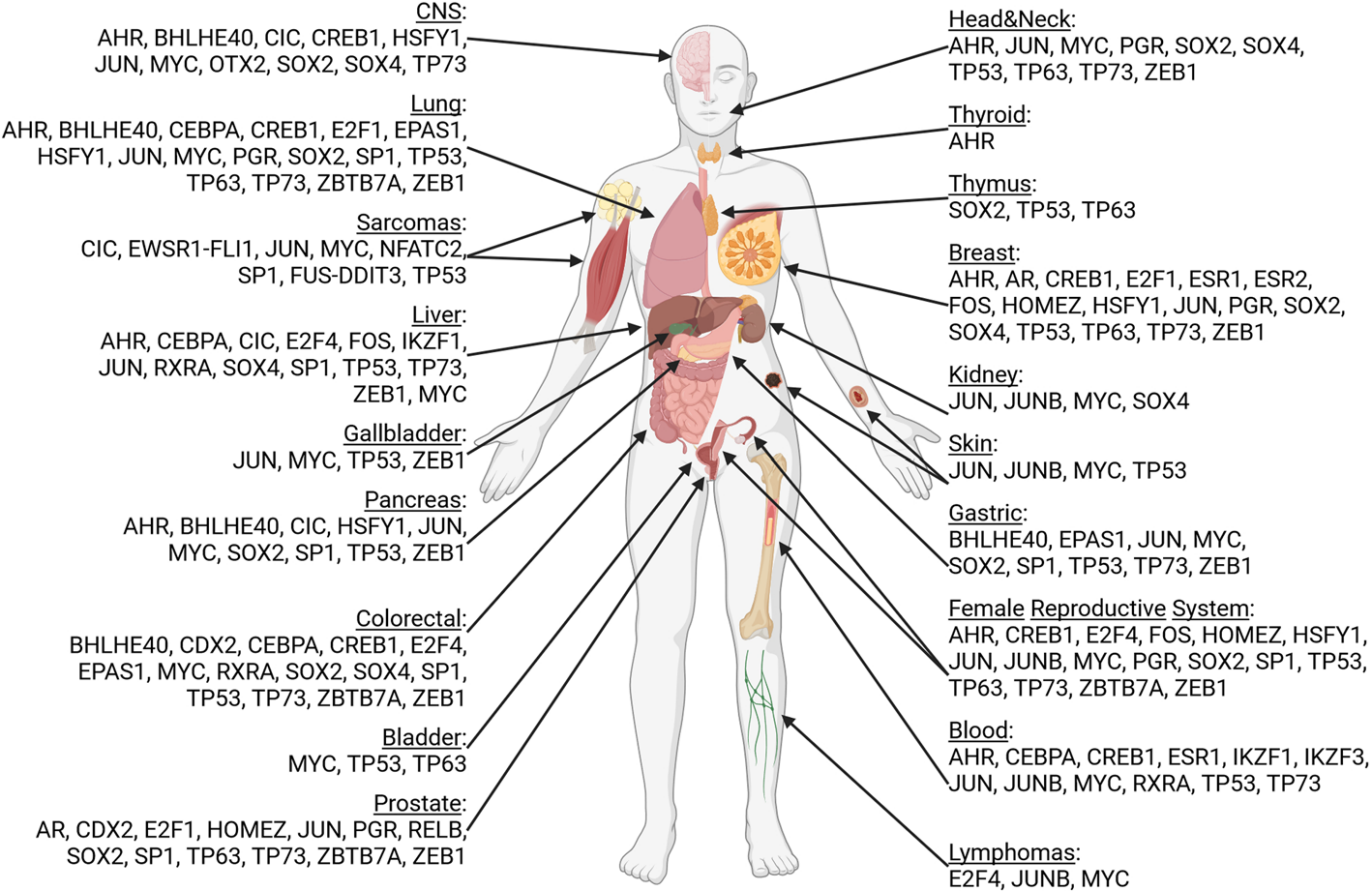
Transcription factors interacting with subunits of SWI/SNF CRCs have a profound role in carcinogenesis

Growth and transformation suppression functions of TP53 are frequently lost in mutant TP53 proteins detected in various cancers. Wild-type TP53 and its mutated derivatives often have different effects on their targets [129]. The gene encoding the vascular endothelial growth factor receptor 2 (*VEGFR2*) was identified as a transcriptional target of mutant TP53 in breast cancer. Different TP53 mutants possess different capacities to activate proto-oncogene *VEGFR2* expression [28, 130], which correlates with decreased survival of patients with breast cancer [131]. Interaction of the SWI/SNF CRCs with mutant TP53 is crucial for *VEGFR2* expression [28]. Mutant TP53 binds near the VEGFR2 promoter, maintaining open chromatin. SWI/SNF regulates numerous mutant TP53-dependent genes, collectively influencing about half of its targets. Mutant TP53-driven VEGFR2 expression is crucial for the enhanced growth and migration of MDA-MB-231 cells. The transcription factor-interacting partners of SWI/SNF CRCs that have a profound role in carcinogenesis are summarized in Fig. 5.

## Effects of SWI/SNF–TF interplay disorder

**Fig. 6:**
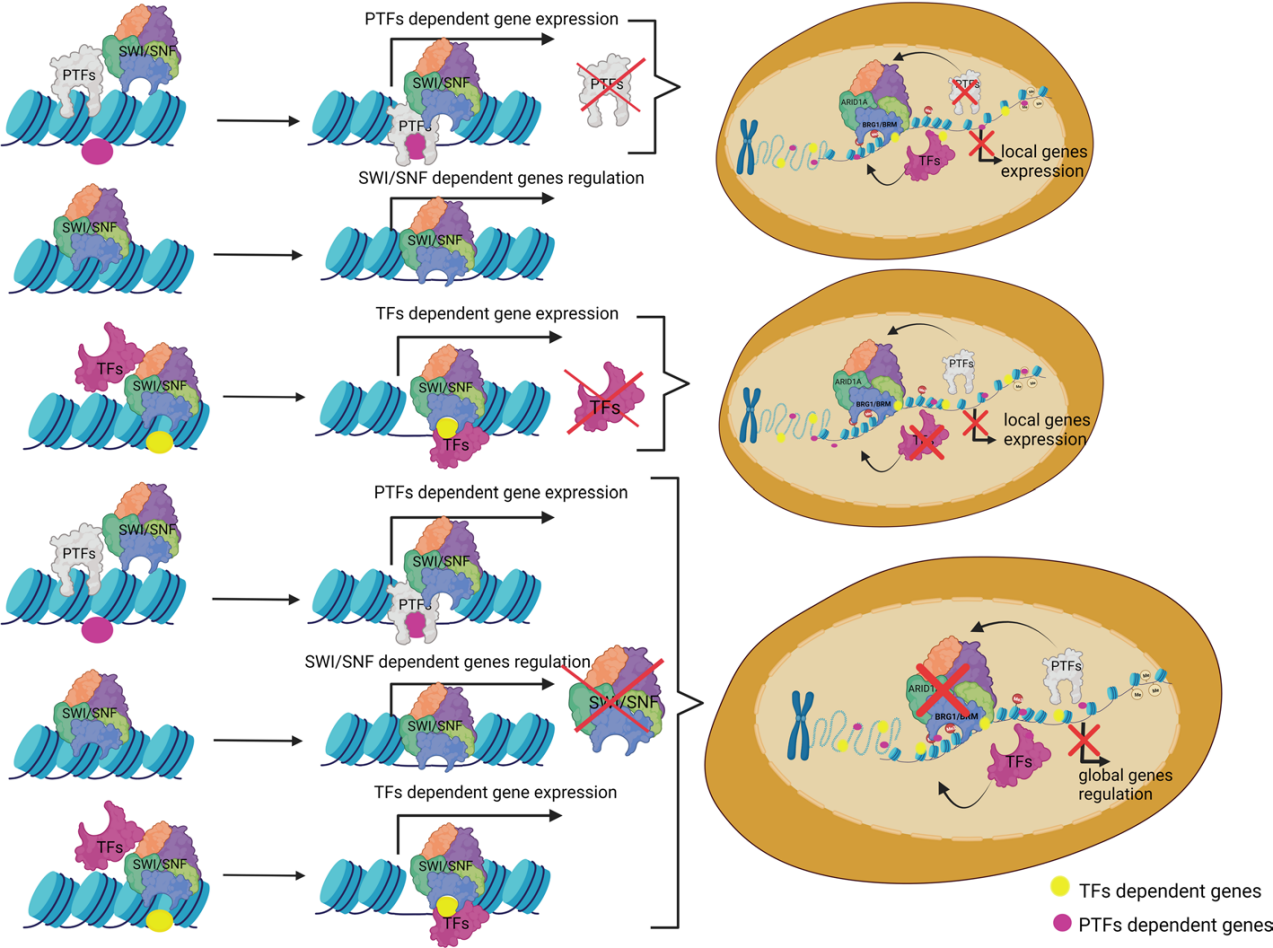
Inactivation of TFs and SWI/SNF CRCs may have various effects. Given their specificity, the inactivation of PTFs and TFs may lead to the altered/impaired expression of PTF- or TF-dependent genes respectively. In contrast, the inactivation of SWI/SNF CRCs may lead to more broad impairment of gene expression in the cell, given their hub-like function, interacting with numerous TFs

TF activation drives proliferation, differentiation, and metabolism, but mutations can disrupt their interaction with chromatin remodelers, deregulating target genes. Similarly, SWI/SNF subunit mutations impair chromatin remodeling, leading to aberrant gene expression. Dysfunction in both TFs and SWI/SNF CRCs can disrupt their interactions, affecting gene regulation and contributing to disease (Fig. 6).

Increasing numbers of TFs [132] and chromatin remodeling complexes components have been implicated in diseases such as Coffin–Siris syndrome (CSS), Nicolaides–Baraitser syndrome (NBS), and autism [133], and are also found in many cancers [31, 133, 134]. Variations in TF DNA binding motifs [135] and TF gene mutations can alter binding specificity or activity, disrupting interactions with DNA and proteins. This may lead to transcriptional deregulation and disease development (Table 1).

**Table 1:** Cancer types and other diseases involving SWI/SNF CRC–TF interplay

SWI/SNF subunit	Involved TF	Disease/cancer type	References
BAF60a	NR3C1 glucocorticoid receptor (GR)	Osteosarcoma (UL3 cell line)	[71]
BAF60a	AR	Prostate carcinoma (LNCaP cell line)	[72]
BRG1	E4F1	Osteosarcoma (U2OS cell line)	[89]
BRG1	SP1	Melanoma (SK-MEL5 cell line)	[98]
BRG1	FUS-DDIT3	Myxoid liposarcoma (MLS 402-91 cell line)	[108]
BRG1	EWSR1-FLI1	Ewing sarcoma (EWS TC-71)	[108]
BAF155 and INI1	DDIT3	Myxoid liposarcoma	[111]
BRM	CEBPA	Human liver tumor samples	[118]
BRG1	PHB	Prostate cancer	[119]
BRG1/BRM	PHB	Human breast cancer (MCF7 and ZR75-1 cell lines)	[70]
BAF60b	IKZF3	Myeloma cell lines	[120]
BRG1	ZEB1	Induction of epithelial-to-mesenchymal transition	[121]
INI1 and BRG1	TP53	Sarcoma (Saos-2 or U2OS cell lines)	[125]
BAF60a	TP53	SW13 and Saos-2 cell lines	[126]
BAF250a	TP53	Ovarian clear cell carcinoma and uterine endometrioid carcinoma (OSE, IOSE-80PC, OVISE, and HEC-1-A)	[121]
BAF155	TP53	Breast cancer (MDA-231 and MDA-468 cell lines)	[23]
BAF53a	TP53	Breast cancer (MDA-468 and SK-BR-3 cell lines)	[23]
BRG1	GR-NR4A2 and GR-HDAC2	Cervix carcinoma (C33A cell line)	[63]
BRM	SIN3A and NR0B2	Hepatoma (HepG2 cell line)	[74]
BRM	CDX2	Colorectal cancer (SW480 and HT-29 cell lines)	[116]
BAF180	MYC	Clear cell renal cell carcinoma (ccRCC)	[136]
INI1	MYC and GLI1	Teratoid rhabdoid tumor	[137]
BAF170 and BAF250b	PCGF1	Embryonal carcinoma (NT2 cell line)	[53]
BAF170	POU5F and DPPA4	Embryonal carcinoma (NT2 cell line)	[53]
BAF250b	NANOG and POU5F	Embryonal carcinoma (NT2 cell line)	[53]
BAF250 and BAF57	PGR	Breast carcinoma (T47D cell line)	[69]
BRM	RB1	Osteosarcoma (U2OS cell line)	[39]
BRG1	RB1-HDAC	Cervical carcinoma (C33A cell line)	[40]
BAF155	GATA4	Human congenital heart malformations	[54]
BAF250a	TBXT and MEF2C	Embryonic stem cells (cardiogenesis)	[55]
BAF250a, BRG1, and BAF170	REST	Embryonic stem cells (neurogenesis)	[55–57]
BAF155 and BAF250a	ASCL1	Neural differentiation (Kolf2C1, pluripotent stem cell line)	[58]

Both aberrations in the integrity of various subtypes of SWI/SNF CRCs, and the activity of TFs, can additionally result in disturbed transcriptional control of target gene expression. For example, in colorectal cancer, mutations in genes encoding both MYC and different SWI/SNF subunit genes have been observed [31, 135]. In clear cell renal cell carcinoma, the BAF180 subunit binds the region of the *RRM2* gene where the target site for the MYC transcription factor is located [136]. An atypical teratoid rhabdoid tumor (ATRT) is caused by loss of the INI1 subunit and has been shown to be linked to aberrant activation of signaling pathways dependent on two TFs–MYC or GLI1 [137]. The engagement of both the SWI/SNF CRCs and TFs in carcinogenesis made these proteins useful targets for synthetic lethality-based cancer therapeutics [134]. For example, LDE225 is the candidate drug against the synthetic lethal target GLI1 (inhibits *GLI1* mRNA expression) in cancers deficient in INI1 [134].

Studying the mechanisms of the regulation of gene expression involving SWI/SNF–TF interactions therefore enables the establishment of novel classes of development of targeted therapies [138].

## Conclusions and future perspectives

**Fig. 7:**
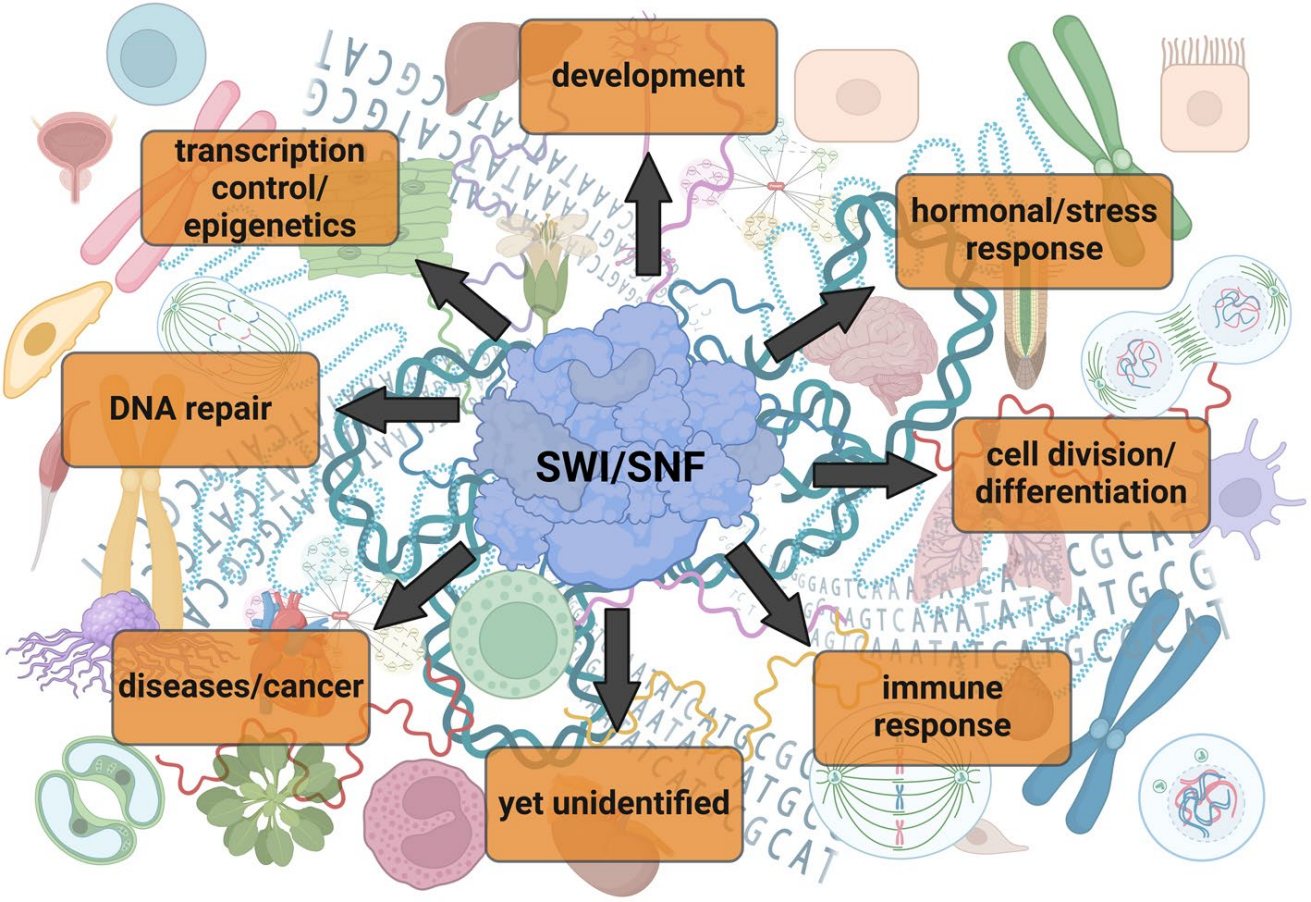
An overview of SWI/SNF CRC–TF interplay-dependent processes in the cell. The TF–SWI/SNF interaction affects numerous processes. Some of them are cell/tissue-specific or dependent on the condition, developmental stage, or disease, among others. Some of such processes are mutually exclusive or play different roles depending on the particular condition or involve certain subtypes of SWI/SNF CRCs. Although there is a large number of identified TFs interacting with SWI/SNF CRCs, there are not yet recognized interactions and/or processes involving such interactions. Therefore, the detailed study identifying further TF–SWI/SNF interactions and dissecting the regulatory role of SWI/SNF–TF interplay requires precise and coordinated research attempts oriented on: the specific process, SWI/SNF subtype, TF type, condition, cell type, and disease, among others

All extracellular and intracellular signals converge on chromatin in the cell nucleus. Although the genetic material is identical in every cell of the organism, different gene profiles are activated depending on the cell or tissue type, creating a unique tissue- and cell-specific landscape. Consequently, a given signal may act differently depending on the tissue or cell type, influenced by various factors, including transcription factors and the SWI/SNF subtypes interacting with them. Therefore, it is crucial to determine which TF interacts with which SWI/SNF subtype and in which tissue or cell type (Fig. 7). Only then can we fully understand the complexity of gene expression regulation and explore its therapeutic modulation. Studies on human SWI/SNF complexes are primarily conducted using cancer cell lines, which have undergone numerous genetic and transcriptomic alterations. Since these complexes are deregulated in most cancers, studying SWI/SNF function in a plant model appears highly attractive. This is especially relevant given that many fundamental mechanisms, SWI/SNF CRCs, and TFs are strongly conserved evolutionarily across kingdoms.

The presented data highlight promising directions for further research into the molecular mechanisms by which TFs and cofactors interact with subtypes of SWI/SNF chromatin remodeling complexes to regulate critical processes. Current knowledge of TF–SWI/SNF interactions remains limited, as most studies focus on subunits common to all subtypes of SWI/SNF complexes, lacking insights into complex-specific interactions [139, 140]. Future research should prioritize examining interactions involving complex-specific subunits to determine their selectivity. Additionally, since TFs and cofactors participate in diverse cellular responses (Supplementary Table 1, Subtables 5–10), further investigation is clearly necessary to explore whether these regulatory interactions extend to other biological processes.

There are only limited reports providing detailed insights into the impact of TF–SWI/SNF interactions on transcription and cellular responses, and such studies have not been extensively continued. Recent high-throughput interactome studies offer valuable global data, enabling the creation of signaling network dependencies. However, most of these studies do not mechanistically explain or experimentally verify the functional influence of these interactions on specific processes. As a result, there is still a lack of studies validating the mechanisms identified in high-throughput research.

This review primarily refers to studies that detail these mechanisms, particularly in humans, while also drawing on data from the evolutionarily conserved functions of SWI/SNF complexes and TFs interactions in *Arabidopsis* and research in model cell lines. However, most of these studies are conducted with cancerous cell lines, leaving a gap in knowledge about corresponding mechanisms in healthy human tissue. The Cancer Genome Atlas (TCGA), which includes genomic, epigenomic, transcriptomic, and proteomic data from both tumor and matched healthy tissue samples, provides an opportunity for systematic tracking of molecular mechanisms involved in gene expression regulation [139, 140]; however, it has still some severe limitations. Therefore, the drawing of solid conclusions on their basis in impossible to the large extent. The data coming from classical genetics study conducted on *Arabidopsis* provide a unique view on the effect of loss of particular SWI/SNF subunits on the whole organism, which is highly advantageous in comparison with research conducted on cancer cell lines with properties already altered by carcinogenesis. The study using *Arabidopsis* clearly indicates that the functions of the SWI/SNF subfamily are specifically diversified between SWI/SNF BAS, MAS, and SAS subtypes in line with emerging data for humans indicative of similar diversification.

Although the function of SWI/SNF CRCs is exhaustively studied, the key questions remain, such as: which TFs, cofactors, SWI/SNF subunits, and subtypes (e.g., cBAF, pBAF, ncBAF in humans or BAS, SAS, MAS in Arabidopsis) control particular processes? Which unidentified, more specific SWI/SNF subtypes interact with specific TFs, and how do these interactions affect processes when disrupted? What chromatin localization, DNA sequences, and interactions with regulatory proteins and chromatin remodeling complexes regulate particular gene expression? Is this interaction tissue-dependent?

Addressing these questions with detailed study will ultimately lead to a better understanding of the interplay between TFs, cofactors, and SWI/SNF complexes across both kingdoms. In addition, it may be crucial for uncovering ancient evolutionary mechanisms that, when disrupted, lead to the development of human diseases. A deeper understanding of how the deregulation of gene expression mechanisms involving the SWI/SNF–TF interplay contributes to disease will be vital for developing novel and more effective therapeutic strategies.

## Supplementary Information

Supplementary Material 1.

## Data Availability

Not applicable.

## Abbreviations

TF: Transcription factor
ATP: Adenosine triphosphate
SWI/SNF: Switch/sucrose nonfermenting
CRC: Chromatin remodeling complex
PTF: Pioneer transcription factor
GTF: General transcription factor
ChIP-seq: Chromatin immunoprecipitation next-generation sequencing
HDAC: Histone deacetylase
SMAD: Mothers against decapentaplegic (family of TFs)
EMT: Epithelial-to-mesenchymal transition
GM-CSF: Granulocyte–macrophage colony-stimulating factor
IL-3: Interleukin 3
PRC1: Polycomb repressive complex 1
MMP2: Matrix metallopeptidase 2
ER: Estrogen receptor
GR: Glucocorticoid receptor
PGR: Progesterone receptor
AR: Androgen receptors
MMTV: Mouse mammary tumor virus
HDL: High-density lipoprotein
ABCA1: ATP binding cassette subfamily A member 1
PRMT5: Type II arginine-specific methyltransferase
CAD: Carbamoyl-phosphate synthetase 2, aspartate transcarbamylase, and dihydroorotase
CYP7A1: Cholesterol 7 alpha-hydroxylase
CYP8B1: Cytochrome P450 family 8 subfamily B member 1
LRH-1: Liver receptor homolog
HR: Homologous recombination
PARP: Poly [ADP-ribose] polymerase
CDK: Cyclin-dependent kinases
siRNA: Small interfering RNA
VEGFR2: Vascular endothelial growth factor receptor 2
TCP: Teosinte branched 1, cycloidea and proliferating cell factor
AP2/ERF: APETALA2/ethylene-response factor
NAC: No apical meristem (NAM), ATAF1/2, cup-shaped cotyledon2 (CUC2)
Dof: DNA binding with one finger
GARP: Golden2, ARR-B, Psr1
SBP: SQUAMOSA promoter-binding protein
EIL: Ethylene insensitive 3-like
ABI3/VP1: Abscisic acid insensitive3/viviparous1
LFY: LEAFY
CrH2H-seq: Cre recombinase yeast two-hybrid sequencing
bHLH: Basic helix–loop–helix
MYB: Myeloblastosis
bZIP: Basic leucine zipper
BAS: BRM-associated SWI/SNF
SAS: SYD-associated SWI/SNF
MAS: MINU-associated SWI/SNF
